# MicroRNA-141-5p Acts as a Tumor Suppressor *via* Targeting RAB32 in Chronic Myeloid Leukemia

**DOI:** 10.3389/fphar.2019.01545

**Published:** 2020-01-22

**Authors:** Jing Bao, Xiaofeng Li, Yuhuan Li, Cheng Huang, Xiaoming Meng, Jun Li

**Affiliations:** ^1^ Anhui Province Key Laboratory of Major Autoimmune Diseases, Anhui Institute of Innovative Drugs, School of Pharmacy, Anhui Medical University, Hefei, China; ^2^ The Key Laboratory of Anti-inflammatory and Immune Medicines, Ministry of Education, Hefei, China; ^3^ Department of Hematology, The First Affiliated Hospital of Anhui Medical University, Hefei, China

**Keywords:** miR-141-5p, chronic myeloid leukemia, RAB32, K652, proliferation

## Abstract

MicroRNA-141-5p (miR-141-5p), an important member of the miR-200 family, has been reported to be involved in cellular proliferation, migration, invasion, and drug resistance in different kinds of human malignant tumors. However, the role and function of miR-141-5p in chronic myeloid leukemia (CML) are unclear. In this current study, we found that the level of miR-141-5p was significantly decreased in peripheral blood cells from CML patients compared with normal blood cells and human leukemic cell line (K562 cells) compared with normal CD34^+^ cells, but was remarkably elevated in patients after treatment with nilotinib or imatinib. Suppression of miR-141-5p promoted K562 cell proliferation and migration *in vitro*. As expected, overexpression of miR-141-5p weakened K562 cell proliferation, migration, and promoted cell apoptosis. A xenograft model in nude mice showed that overexpression of miR-141-5p markedly suppressed tumor growth *in vivo*. Mechanistic studies suggested that RAB32 was the potential target of miR-141-5p, and silencing of RAB32 suppressed the proliferation and migration of K562 cells and promoted cell apoptosis. Taken together, our study demonstrates that miR-141-5p plays an important role in the activation of K562 cells *in vitro* and may act as a tumor suppressor *via* targeting RAB32 in the development of CML.

## Introduction

Chronic myeloid leukemia (CML) is an acquired bone marrow proliferative malignant tumor. Philadelphia chromosome (Ph), which is one of the cytogenetic characteristics of CML, arose from a translocation between chromosome 9 and chromosome 22 (t [9]; [22] [q34; q11]). This exchange forms a fusion gene called BCR–ABL on chromosome 22, which results in tyrosine kinase deregulation (Patnaik et al., 2014). Imatinib is a small molecule drug that is used in CML treatment, and it is BCR–ABL tyrosine kinase inhibitor (TKI). Imatinib results in a hematological complete remission rate of 95% in CML. However, side effects and drug resistance to TKI lead to poor efficacy in some patients (Jabbour and Kantarjian, 2014). Therefore, it is particularly urgent to examine the pathogenesis of CML and seek new therapeutic strategies. Previous studies confirmed that multiple microRNAs (miRNAs) played vital roles in CML.

MicroRNAs (miRNAs) are small (18–22 nucleotides) non-coding RNAs that widely exist in prokaryotic and eukaryotic cells. MiRNA specifically binds to its target gene *via* the 3-untranslated region (3′-UTR), which is located at one end of the mRNA, to regulate gene expression and plays a vital role in many biological processes (Spizzo et al., 2009). The miRNA200 family includes five different members: miR-200a, miR-200b, miR-200c, miR-141, and miR-429. The expression and role of the miR-200 family are different in diverse cell environments, including gastric cancer (Zhou et al., 2015), breast cancer (Hilmarsdottir et al., 2014), lung cancer (Kim et al., 2015), and brain cancer (Men et al., 2014). Notably, San Jose-Eneriz et al. (2009) found that the level of miR-141 was decreased in drug-resistant CML patients. However, the biological effect and function of miR-141 in CML remain unclear.

The RAB Protein Is the largest subfamily of the Ras superfamily, which are also known as small Gtpases (Pereira-Leal and Seabra, 2000). Most RAB proteins play an important role in regulating membrane transport and signaling (Prashar et al., 2017). Haile et al. (2017) found that RAB32 was located in mitochondria, and it was closely related to mitochondrial function. Notably, bioinformatics analysis predicted that RAB32 was the potential target of miR-141-5p. However, the function and potential mechanism of miR-141-5p targeting of RAB32 in CML remain poorly understood.

The present study observed the function of miR-141-5p in CML K562 cells and patients to elucidate its underlying mechanisms in CML tumorigenesis. Our results may provide new clues for CML diagnosis and targeted therapies.

## Materials and Methods

### Human Peripheral Blood Specimen Collection

The study included 21 cases with a recent diagnosis of CML who presented in the chronic phase to the Department of Hematology, the First Affiliated Hospital of Anhui Medical University, Hefei, China from April 2015 to September 2016. Fourteen healthy controls were also recruited from the medical examination center of the same hospital. The basic information of the CML patients is shown in **Table 1**. The Medical Ethics and Human Clinical Trial Committee of Anhui Medical University approved the experiment. All of the research subjects volunteered to donate their blood samples for the research. We immediately stored these blood samples at −80°C. The peripheral blood specimens acquired preconditions with human peripheral blood lymphocyte separation fluid (Tianjin Hao Yang, China) before RNA extraction and protein analysis. The procedure was based on the manufacturer’s protocol.

**Table 1:** Basic information of the CML patients.

Characteristic	Value
Median age, years (range)	55.6 (20–74)
Sex	
Female	12
Male	9
Median bone marrow blast, % (range)	3.8 (1–6.8)
Median WBC count (×10^9^/L) (range)	82.6 (19.3–332.1)
Median hemoglobin (g/L) Median BCR–ABL, % (range)	100.9 (80.2–133.8) 75.9 (44.9–202.1)

WBC, white blood cell.

### Materials and Reagents

Cell cultures for K562 cells and K562/G cells included Bioind (BI) from Biological Industries (Bioind, Israel) and RPMI1640 medium from Hyclone (Logan, Utah, USA). Primary antibodies included the following monoclonal antibodies: rabbit-anti-c-Myc and rabbit-anti-cyclin D1 provided by Cell Signaling (Danvers, MA, USA); rabbit-anti-MMP-9 purchased from Millipore (Billerica, MA, USA); and rabbit-anti-MMP-3 and rat-anti-β-actin from Bioworld (Shanghai, China). A goat anti-RAB32 polyclonal antibody (No: ab192459, Lot: GR267679-1) was purchased from Abcam (Cambridge, UK). We chose goat anti-rabbit immunoglobulin horseradish peroxidase (bioss, Beijing, China) as the secondary antibody. C-Myc, cyclin D1, matrix metalloproteinases 3 and 9 (MMP-3, MMP-9), RAB32, and β-actin primers were purchased from Sangon Biological and Technological Company (Shanghai, China).

### Cell Culture

We used K562 cells and K562/G cells for *in vitro* experiments, and these cell lines were purchased from the Institute of Hematology, Chinese Academy of Medical Sciences (Tianjin, China). The complete medium contained RPMI-1640 medium (Hyclone, USA), 10% (v/v) heat-inactivated BI (Bioind, Israel) and a 1% penicillin and streptomycin mixture (Beyotime, China). Cells were seeded in culture flasks at appropriate concentrations and grown in an incubator (37°C, 5% CO_2_).

### Animal Experiments

Twenty four-week-old female BALB/c nude mice from Lingchang Biotechnology Co. Ltd. (Shanghai, China) were used to analyze CML tumorigenicity *in vivo*. The Animal Care and Use Committee of Anhui Medical University, China approved the animal experiments. Twenty nude mice were randomly divided into two equal groups (experimental group and control group). K562 cells (1×10^7^) were infected with miR-141-5p mimics or the negative control (NC) lentivirus prior to subcutaneous inoculation into the right sub axillary region of each nude mouse. The major diameters (L) and minor diameters (W) of the transplanted tumors were measured accurately every 3 days. We calculated the tumor volumes using the formula: V = π/6 × L × W × W. Twenty-one days after the above treatment, the mice were euthanized to determine the final weights and volumes of the transplanted tumors. All mice were sacrificed *via* cervical dislocation.

### Lentiviral MiR-141-5p Construction

The lentiviral vector system from Genechem (Shanghai, China) selected in this experiment contained three plasmids: GV209, pHelper 1.0, and pHelper 2.0 vector. The GV209 lentiviral vector contains 5′-LTR and 3′-LTR, the basic components of HIV, and other auxiliary components. The pHelper 1.0 vector expresses the major structural proteins encoding the virus, specific enzymes, and regulatory factors required for gene expression. Genes for virus packaging virus are included in the pHelper 2.0 vector. We obtained specially designated lentiviral particles with miR-141-5p mimics/NC *via* modification of the GV lentiviral vector before virus packaging in the 293T cells. Three plasmids (GV209, pHelper 1.0, and pHelper 2.0 vector) were compounded carefully using reagent from Genechem (Shanghai, China) according to the manufacturer’s instruction. The cells were incubated at room temperature for 15 min. We cotransfected three plasmids into 293T cells using lipofectamine 2000, and cells were cultured with complete DMEM medium (including 10% BI, 1% antibiotic mixture) in the incubator (37°C, 5% CO_2_) for 48–72 h. The virus was harvested, concentrated, and purified *via* centrifugation (4,000×g, 10 min, 4°C). Impurities were removed *via* filtration through a 0.45 µm filter followed by centrifugation (25,000 rpm 4°C) for 2 h. The virus deposit was collected and preserved in a −80°C refrigerator.

### Plasmid Construction

RAB32-N1 (contains 3′-UTR) and empty-N1 plasmid intended for plasmid construction were purchased from Genechem (Shanghai, China). K562 cells were co-transfected with the recombined vector (containing miR-141-5p mimics and RAB32-N1 plasmid) using Lipofectamine™ 2000, and these cells were regarded as the experimental group. The cells co-transfected with miR-141-5p mimics and empty plasmid were the control group, according to the manufacturer’s manuals. We also set a blank control to control variable.

### Cells Transient Transfection

The following oligonucleotide sequences pairs were designed: MiR-141-5p-inhibitor: 5′-UCCAACACUGUACUGGAAGAUG-3′; MiR-141-5p-mimics: Sense: 5′-CAUCUUCCAGUACAGUGUUGGA-3′, Antisense: 5′-CAACACUGUACUGGAAGAUGUU-3′; and RAB32-siRNA: Sense: 5′-GGACCAAUUCUGCAAAGAATT-3′, Antisense: 5′-UUCUUUGCAGAAUUGGUCCTT-3′. A negative interfering siRNA (Gene Pharma, Shanghai, China) was used in parallel as the control group. We replaced Opti-MEM (Serum-free medium) for the K562 cells the day before transfection and cultured cells at 37°C, 5% CO_2_ overnight. Before transfecting, we mixed Lipofectamine 2000 and Opti-MEM and placed the mixture for 5 min at room temperature. MiR-141-5p-mimics/inhibitor/siRNA/NC was added into Opti-MEM separately followed by incubation at room temperature for 5 min. We obtained the RNA/Lip2000 compounds for transfection of the K562 cells *via* mixing diluted Lipofectamine 2000 and mimics/inhibitor/siRNA/NC followed by incubation for 30 min. Cells were incubated in complete RPMI1640 medium in 37°C, 5% CO_2_ for 6 to 8 h and cultured for 48 h for further detection, including q-PCR, Western blotting, and flow cytometry analysis (FACS).

### Cells Cycle Analysis

We fixed the K562 cells in 70% ethanol (4°C, overnight) after a 48-h transfection with miR-141-5p inhibitor, miR-141-5p mimics, RAB32-RNAi, and RAB32-N1. The cells were collected and centrifuged (1,000×g, 5 min). We used PBS to wash the ethanol and surplus transfect, and centrifuged (1,000×g, 3 min) twice. The supernatant was discarded. PI staining buffer (Beyotime, China) was added, and the solutions were sheltered from light for 30 min at room temperature prior to flow cytometry (FACS). A Beckman Coulter flow cytometer was used to analyze the intracellular DNA content. All procedures were strictly performed according to the kit instructions.

### Cells Apoptosis Analysis

Similar to the pretreatment of cell cycle analysis, K562 cells were fixed and washed. We used an Annexin-V-FITC apoptotic detection kit (Best Bio, China) to analyze the cell apoptosis ratio. We added Annexin-V-FITC to the cell suspension with Annexin-V binding buffer and incubated (light-shielded) the cells at 2–8°C for 15 min. We added PI to the suspension and incubated for another 5 min prior to flow cytometry (FACS) with a Beckman Coulter flow cytometer immediately. All procedures referred to the kit instructions.

### Quantitative Real-Time PCR

To determine the expression levels of miR-141-3p and 5p, we extracted total RNA from K562 cells using RISO RNA Isolation Reagent (Biomics, USA) based on the manufacturer’s instructions. We used EzOmics One-Step qPCR Kit (Biomics, USA) and miRNA qPCR Detection Primer Set (Biomics, USA). The QuantiFast SYBR Green RT-PCR kit (QIAGEN, Germany) was used to determine the various mRNA levels of C-Myc, cyclin D1, MMP-3, MMP-9, RAB32, and β-actin. The following sequences of primer pairs were used: c-Myc (forward: 5′-GGACTATCCTGCTGCCAAGA-3′, reverse: 5′-CGCCTCTTGACATTCTCCTC-3′); cyclinD1 (forward: 5′-GATCAAGTGTGACCCGGACT-3′, reverse: 5′-TCCTCCTCTTCCTCCTCCTC-3′); MMP-3 (forward: 5′-GGCCAGGGATTAATGGAGAT-3′, reverse: 5′-TGAAAGAGACCCAGGGAGTG-3′); MMP-9 (forward: 5′-GTACCACGGCCAACTACGAC-3′, reverse: 5′-GCCTTGGAAGATGAATGGAA-3′); RAB32 (forward: 5′-AGCTGTTGGTGCTTTTGTAGTC-3′, reverse: 5′-GCTGCCATTTGGAAGATGAA-3′); β-actin (forward: 5′-GCCAACACAGTGCTGTCTGG-3′, reverse: 5′-AGGAGGAGCAATGATCTTG-3′). The following reaction program of PCR was set: thermal denaturation for 1 cycle (95°C, 10 min) and amplification for 40 cycles (95°C for 20 s, 62°C for 30 s, 72°C for 30 s). The last step was analysis of the dissolution curve, and we used the 2^−△△CT^ method (β-actin as internal parameter) to analyze the relative quantitative real-time PCR results.

### Western Blotting

Total proteins of K562 cells were abstracted using RIPA protein lysate containing a Roche protease inhibitor. Proteins were diluted to working concentration in SDS-PAGE loading buffer. SDS-PAGE was used to separate the extracted proteins based on molecular weights, and PAGE gel areas were selected for transmembrane proteins according to the relative molecular weights of c-Myc, cyclinD1, MMP-3, and MMP-9. These proteins were transferred onto PVDF membranes (Millipore, USA), which were blocked with 5% skim milk dissolved in TBST for 2–3 h at room temperature (or overnight at 4°C). The nitrocellulose blots were washed three times with TBST before incubation with the diluted primary antibodies (1:500) overnight at 4°C. Secondary antibodies (1:1,000 goat horseradish peroxidase anti-mouse or anti-rabbit IgG: 5% skim milk) were used, and the membranes were incubated on a shaker for 2 h at room temperature (or overnight at 4°C). After washing with TBST buffer for three times, the protein levels were detected from the blots by using ChemiDocTM MP Imaging System (Bio-Rad).

### Luciferase Reporter Gene Assays

K562 cells were transplanted into a 24-well plate in advance. K562 cells were co-transfected plasmids and interferences using Lipofectamine 2000. We chose RAB32-3′-UTR-WT plasmid gene (200 ng), miR-141-5p mimics or NC control (60 nmol) (Gene Pharma, Shanghai), 2.25 μl Lipofectamine 2000, and 100 μl Opti-MEM (Invitrogen, USA) to detect the results. The following sequences of primer pairs were used: Rab32-3′UTR WT (forward: 5′-GATAACATAAACATAGAGGAAGCTGCC-3′; reverse: 5′-GGTGGTAATAAAATGTTACCTCCAGTC-3′). Cells were harvested after 48 h of transfection and lysed. We used the Dual-Luciferase Reporter Assay (Promega, USA) to measure the luciferase activities.

### Statistical Analysis

The data were analyzed using SPSS16.0 software. We used single factor analysis of variance and Dunnett’s test. All data are expressed as the means ± SD, and *P* < 0.05 indicated statistical significance.

## Results

### MiR-141-3p and 5p Were Low-Expression in CML

**Figure 1:**
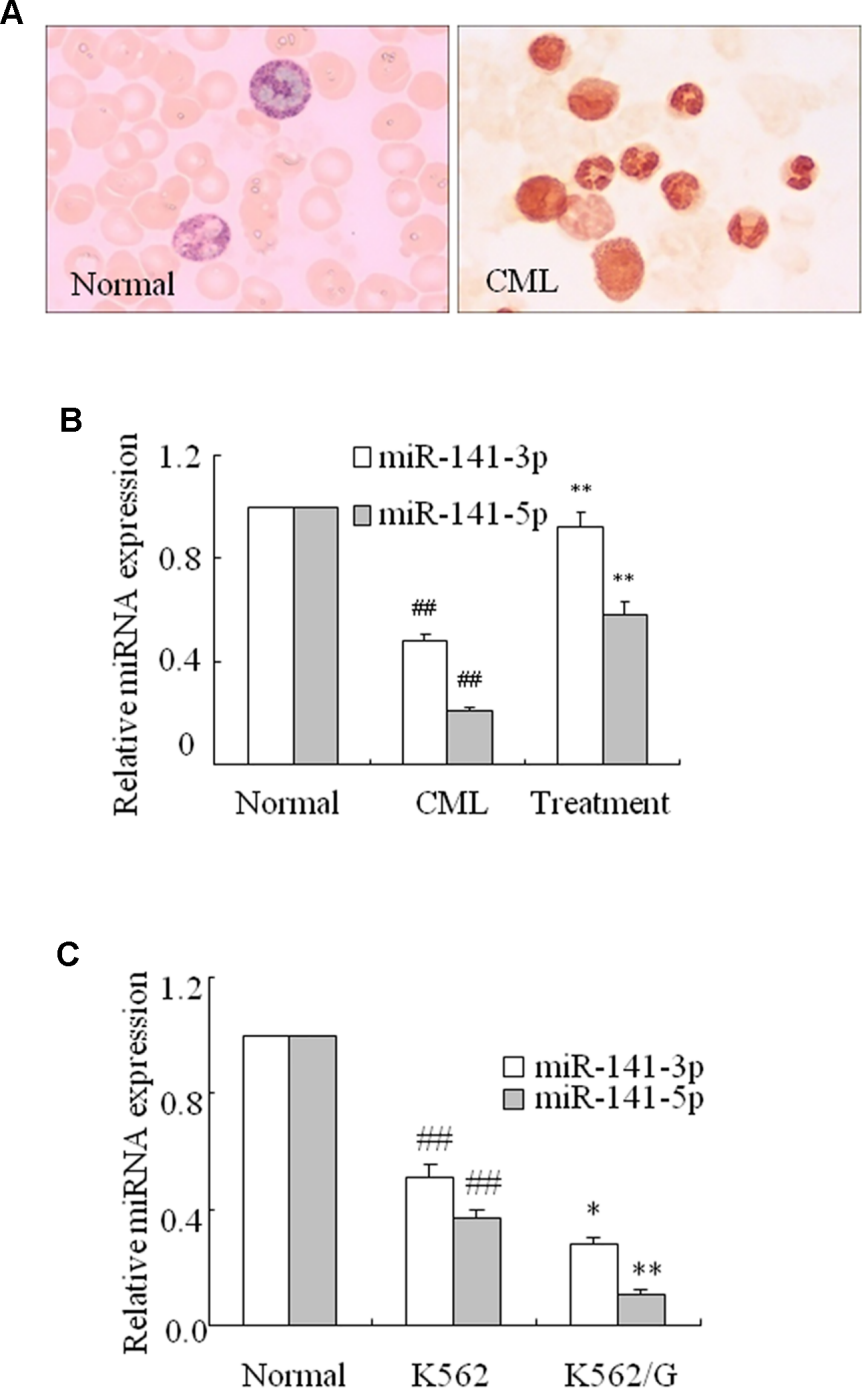
miR-141-3p and miR-141-5p exhibited low expression in fresh CML samples. **(A)** Neutrophil alkaline phosphatase decreased remarkably in peripheral blood in CML and normal patients (×100). **(B)** The levels of miR-141-3p and miR-141-5p were determined using q-PCR in normal, CML and treated patients (^##^ *P* < 0.01 vs. normal group, **P < 0.01 vs. CML group). **(C)** The levels of miR-141-3p and miR-141-5p were analyzed using q-PCR in K562 and K562/G cells. All data are expressed as the means ± SD (^##^ *P* < 0.01 vs. normal CD34^+^ cells, *P < 0.05, **P < 0.01 vs. K562).

MiR-141-3p and miR-141-5p levels were assessed using q-PCR in CML. Alkaline phosphatase staining (**Figure 1A**) confirmed that the neutrophil alkaline phosphatase score was remarkably decreased in the peripheral blood of CML compared with healthy samples. Furthermore, the levels of miR-141-3p and 5p were significantly decreased in CML compared with healthy samples. Eighteen of the 21 newly diagnosed CML patients were treated with nilotinib or imatinib. The remaining three patients did not receive treatment and were subsequently lost to follow-up. Interestingly, these two miRNAs were remarkably upregulated in CML patients at 3 months after treatment with nilotinib or imatinib (**Figure 1B**). Moreover, compared with healthy controls’ normal CD34^+^ cells, the levels of miR-141-3p and 5p in human leukemic cell line K562 and K562/G cells were markedly downregulated. These two miRNAs were more significantly decreased in K562/G cells than that of K562 cells (**Figure 1C**). K562/G is an imatinib-resistant K562 cell line. These results revealed that miR-141-3p and -5p exhibited low expression in CML. MiR-141-5p was further selected in this study because miR-141-5p was significantly lower in CML patients.

### Inhibition MiR-141-5p Enhanced K562 Cell Proliferation and Migration Abilities

**Figure 2:**
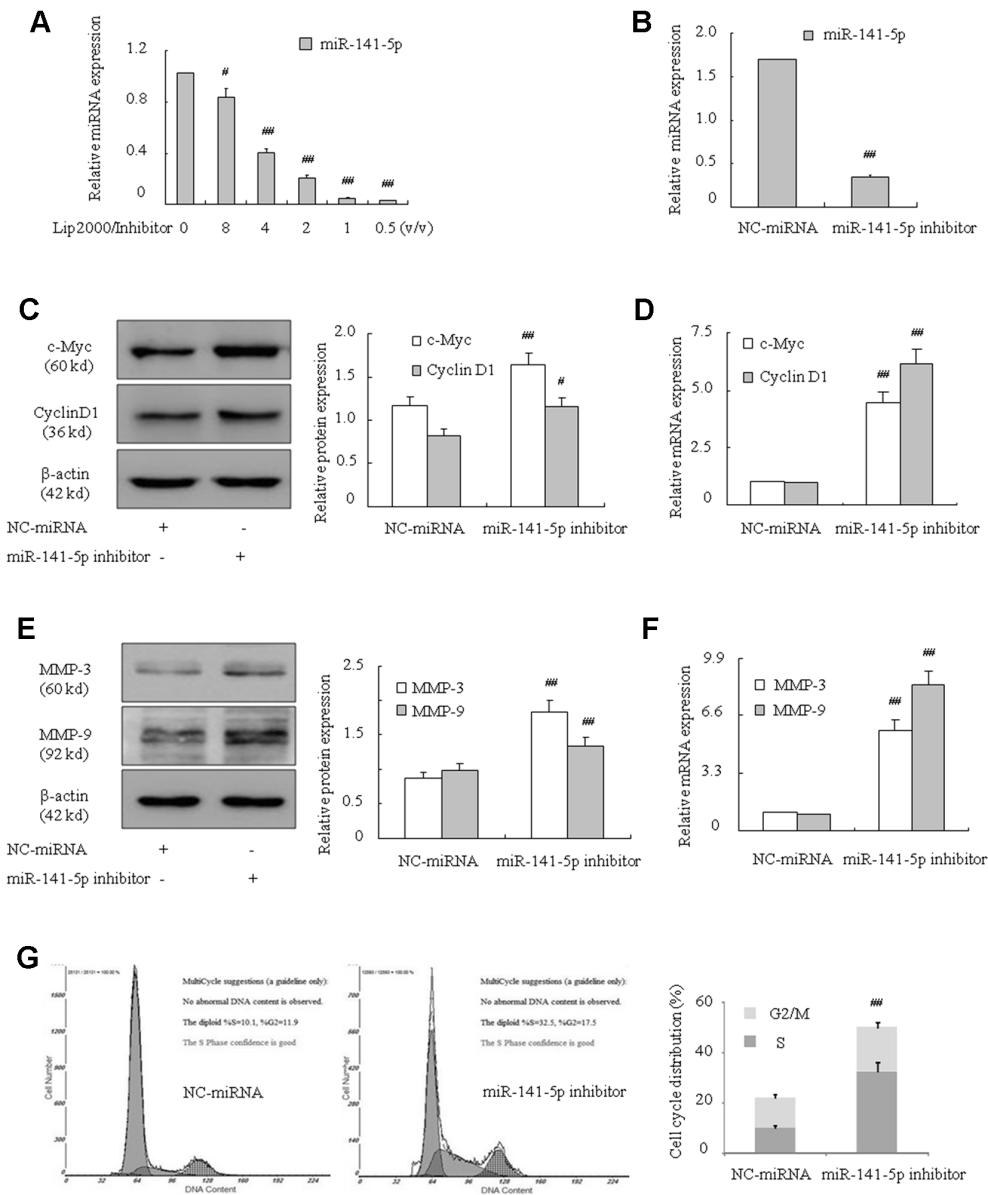
Effect of miR-141-5p inhibitor increased the proliferation and migration of K562 cells. **(A)** q-PCR was used to detect the transfection efficiency of the miR-141-5p inhibitor. MiR-141-5p expression was influenced by the concentration gradient of Lip2000 and miR-141-5p inhibitor mixture in K562 cells. **(B)** The level of miR-141-5p was tested using q-PCR in K562 cells treated with the most appropriate concentration of the miR-141-5p inhibitor. **(C)** The protein contents of c-Myc and cyclin D1 were detected using Western blotting in K562 cells with an miR-141-5p inhibitor compared to the control group. **(D)** The mRNA expression levels of c-Myc and cyclin D1 were tested using q-PCR in K562 cells treated with an miR-141-5p inhibitor or negative control separately. **(E)** The protein contents of MMP-3 and MMP-9 were detected using Western blotting in K562 cells treated with the miR-141-5p inhibitor or negative control. **(F)** q-PCR was used to reveal the mRNA expression of MMP-3 and MMP-9 in K562 cells treated with the miR-141-5p inhibitor or negative control. **(G)** Cell cycle of K562 cells was assessed using FACS analysis after incubation with the miR-141-5p inhibitor for 48 h. All data are expressed as the means ± SD (^#^ *P* < 0.05, ^##^ *P* < 0.01 vs. NC group).

To further identify the influence of miR-141-5p, we assessed the effect of miR-141-5p on the proliferation and migration of K562 cell lines *via* transfection with miR-141-5p inhibitor. Firstly, the downregulation of miR-141-5p in K562 cells were verified using q-PCR (**Figures 2A, B**). As shown in **Figures 2C–F**, the oncogene c-Myc, cyclin D1, MMP-3, and MMP-9 mRNA and protein levels were significantly upregulated after transfection with miR-141-5p inhibitor. Furthermore, we found that the ratios of cells in S and G2/M phases were increased after transfection with the miR-141-5p inhibitor (**Figure 2G**). These results demonstrated that the miR-141-5p inhibitor remarkably enhanced K562 cell proliferation and migration abilities.

### Overexpression of MiR-141-5p Weakened K562 Cell Proliferation and Migration Abilities and Enhanced Apoptosis

**Figure 3:**
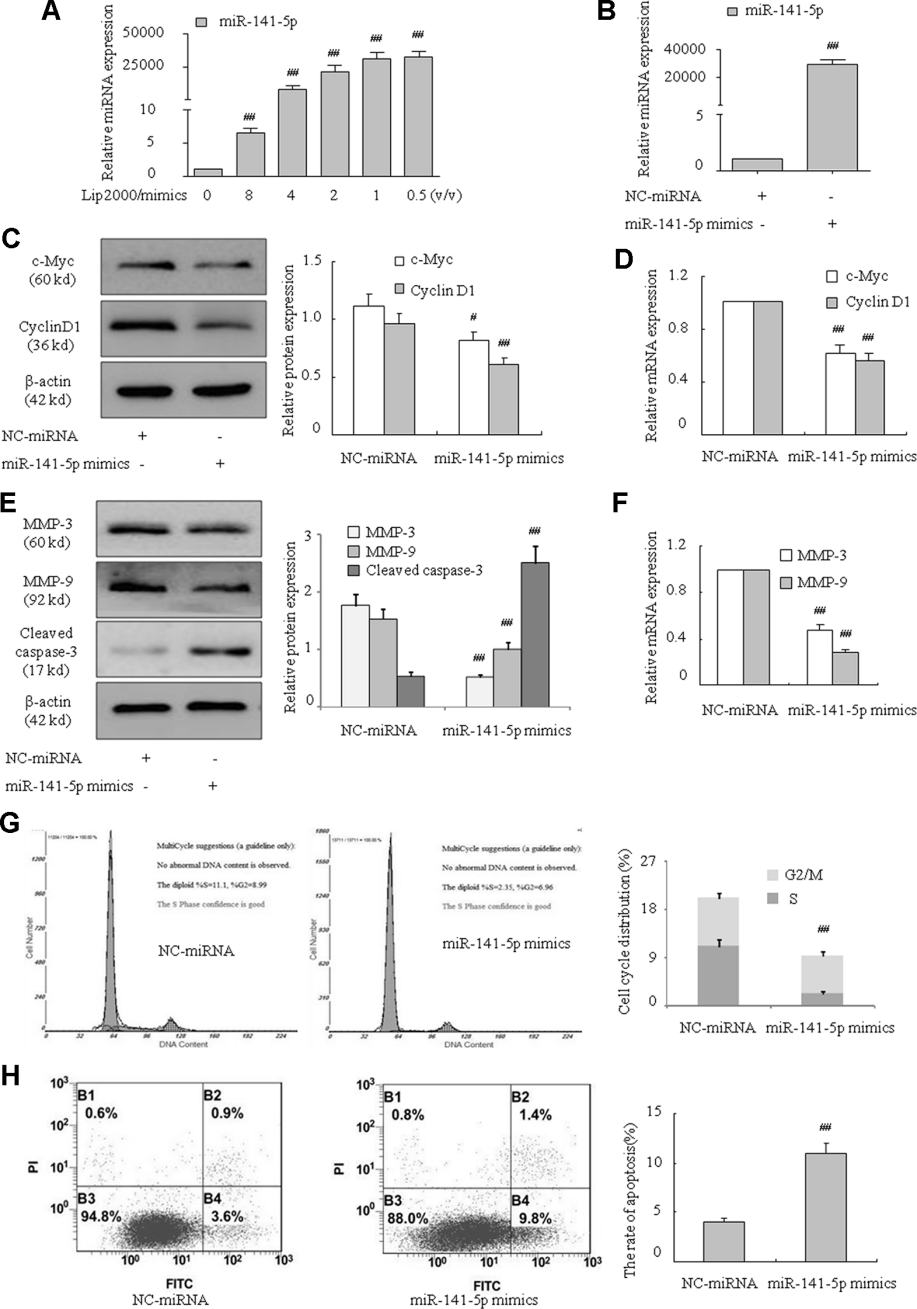
MiR-141-5p mimics inhibited proliferation and migration and promoted apoptosis in K562 cells. **(A)** Concentration-dependent mimic expression of miR-141-5p by Lip2000 and miR-141-5p mimics in K562 cells was detected using q-PCR. **(B)** The level of miR-141-5p was tested using q-PCR in K562 cells with miR-141-5p mimics. **(C)** The protein contents of c-Myc and cyclin D1 in K562 cells with miR-141-5p mimics were determined using Western blotting. **(D)** The mRNA expression of c-Myc and cyclin D1 were detected using q-PCR in K562 treated with miR-141-5p mimic or negative control. **(E)** The protein contents of MMP-3, MMP-9, and cleaved caspase-3 in K562 cells with miR-141-5p mimics or the NC group were determined using Western blotting. **(F)** The mRNA expression of MMP-3 and MMP-9 were detected using q-PCR in both groups. **(G)** Cell cycle of K562 cells was assessed using FACS analysis after incubation with miR-141-5p mimics for 48 h. **(H)** Cell apoptosis analyses of K562 cells treated with miR-141-5p mimics were performed using FACS. All data are expressed as the means ± SD (^#^ *P* < 0.05, ^##^ *P* < 0.01 vs. NC group).

MiR-141-5p mimics were used to further verify the effect of miR-141-5p on the proliferation, migration, and apoptosis of K562 cells. K562 cells were treated with miR-141-5p mimics for 48 h, and miR-141-5p level was remarkably increased compared to control (**Figures 3A, B**). As expected, c-Myc, cyclin D1, MMP-3, and MMP-9 mRNA and protein levels were significantly decreased after overexpression of miR-141-5p (**Figures 3C–F**). On the contrary, the cleaved caspase-3 level was increased (**Figure 3E**). Flow cytometric analysis showed that overexpression of miR-141-5p resulted in an obvious decrease in the proportions of cells in S and G2/M phases (**Figure 3G**). Annexin V-FITC/PI staining was used to detect whether overexpression of miR-141-5p induced cell apoptosis. As showed in **Figure 3H**, miR-141-5p mimics induced the overexpression of miR-141-5p, which enhanced apoptosis of K562 cells. These results strongly indicated that overexpression of miR-141-5p weakened the proliferation and enhanced K562 cell apoptosis. Therefore, miR-151-5p may be involved in K562 cell proliferation, migration, and apoptosis and play a vital role in the pathogenesis of CML.

### Upregulation of MiR-141-5p Suppressed Tumor Xenograft Growth in Nude Mice

**Figure 4:**
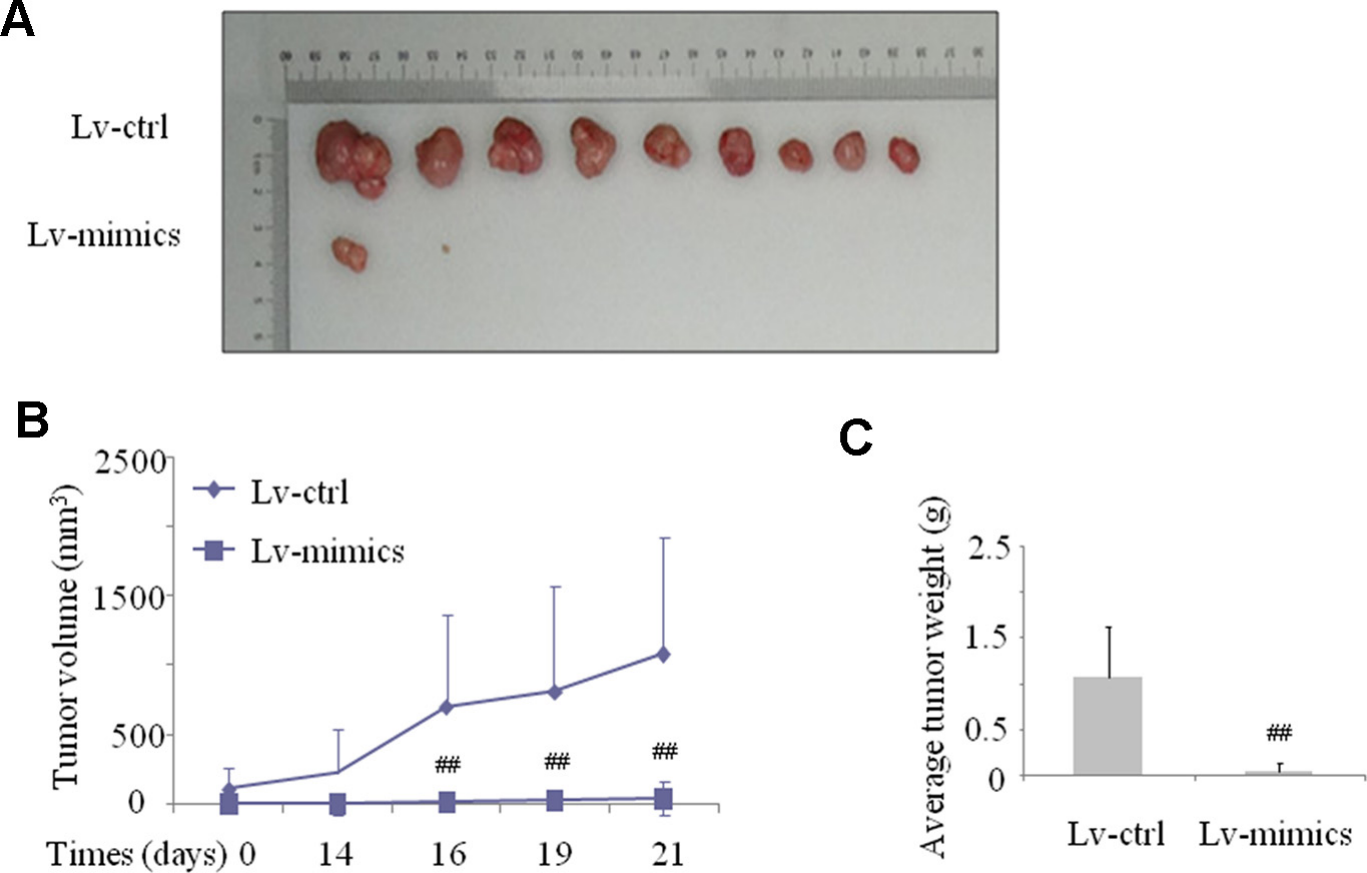
Upregulation of miR-141-5p suppressed tumor xenograft growth in nude mice. **(A)** The graphs are representative images of the tumors isolated 3 weeks after inoculation. **(B)** The growth curves of transplanted tumors were established *via* measurement of tumor volumes every three days. **(C)** The growth curves of transplanted tumors were analyzed by weight. All experimental data are expressed as the means ± SD (^##^ *P* < 0.01 vs. NC group).

As described in previous results, we demonstrated that miR-141-5p expression was lower in CML patients and K562 cell lines. Next, we wonder the underlying effect of miR-141-5p *in vivo*. We established a K562 cell line that stably expressed the miR-141-5p by transfecting with a lentiviral plasmid and inoculated female nude mice subcutaneously. The volumes and weights of transplanted tumors were observed and measured every third day. Consistent with the cell growth results *in vitro*, tumor growth was significantly slower in the miR-141-5p mimics group than the control group (**Figure 4A**). As shown in **Figure 4B**, the tumor volumes were observably decreased in the miR-141-5p mimics group after 21 days. The average tumor weights of the miR-141-5p mimics group were also reduced compared to the control (**Figure 4C**). Overall, these results indicated that the upregulation of miR-141-5p suppressed tumor growth *in vivo*, which may be related to K562 cell proliferation, migration, and apoptosis and play a vital role in the tumorigenesis of CML.

### RAB32 Was a Target Gene of MiR-141-5p

**Figure 5:**
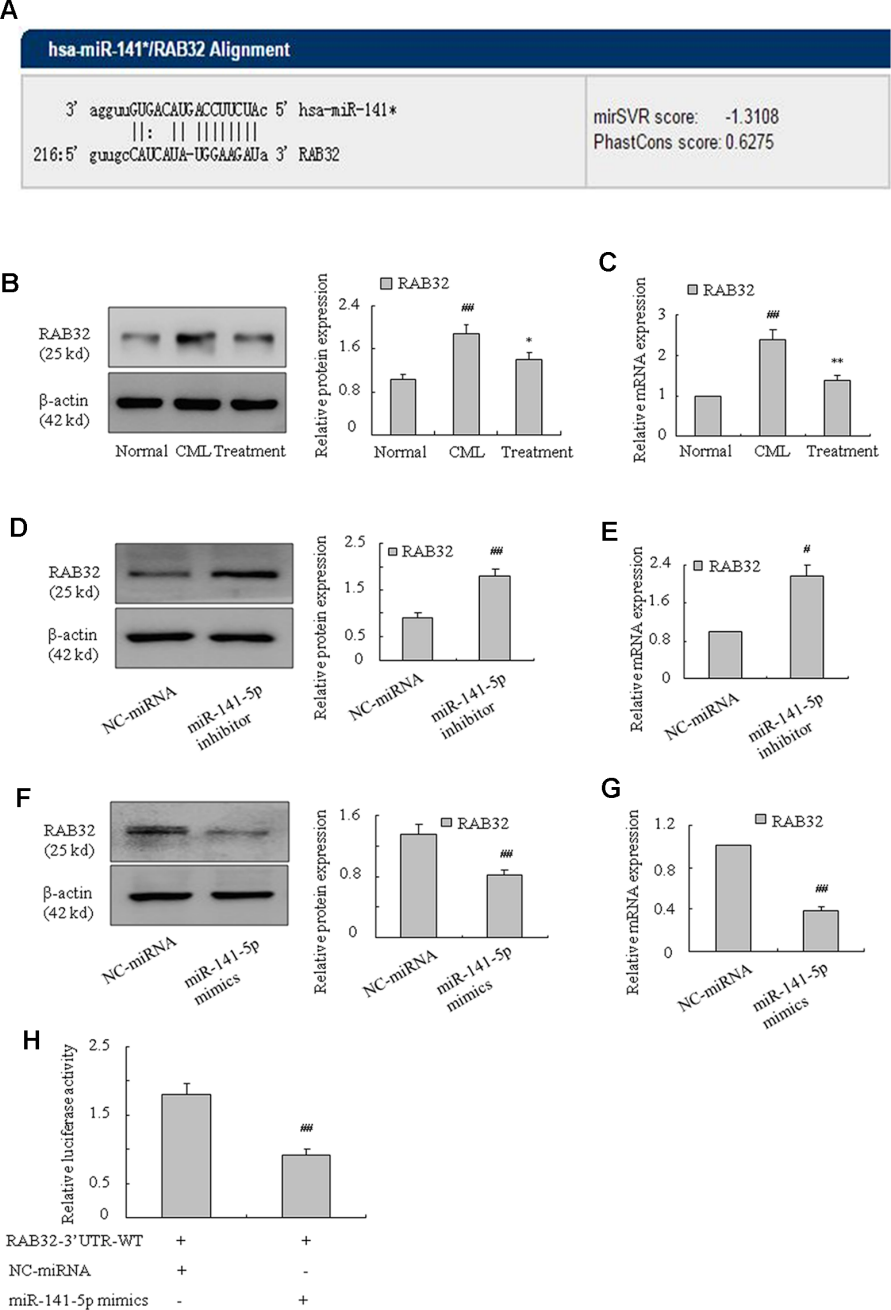
RAB32 is a direct target of miR-141-5p. **(A)** Bioinformatics analyses predicted that miR-141-5p was complementary to 3′ non-coding regions of RAB32 mRNA. **(B)** The protein expression of RAB32 was measured using Western blotting in normal, CML, and treated patients separately (^##^ *P* < 0.01 vs. normal group, ^*^ *P* < 0.05 vs. CML group). **(C)** The mRNA expression of RAB32 was detected using q-PCR in normal, CML, and treatment patients (^##^ *P* < 0.01 vs. normal group, ^**^ *P* < 0.01 vs. CML group). **(D)** Western blotting was used to determine the protein level of RAB32 in K562 cells with the miR-141-5p inhibitor. **(E)** The mRNA expression of RAB32 was detected using q-PCR in K562 cells with the miR-141-5p inhibitor. **(F)** RAB32 protein level in K562 cells with miR-141-5p mimics was determined using Western blotting. **(G)** q-PCR was used to analyze the RAB32 mRNA expression in K562 cells pretreated with miR-141-5p mimics. **(H)** RAB32 3′ UTR and miR-141-5p mimics in K562 cells were tested using the dual luciferase assay. All data are expressed as the means ± SD (^#^ *P* < 0.05, ^##^ *P* < 0.01 vs. NC group).

To further examine the underlying mechanism of miR-141-5p in K562 cells, we used bioinformatics analysis (miRanda, miRbase Targets, and TargetScan) to predict the possible targets of miR-141-5p. These three approaches predicted that miR-141-5p was complementary to 3′ non-coding regions of RAB32 mRNA (**Figure 5A**). We found that the level of RAB32 was higher in CML samples than healthy samples, and it was significantly downregulated in patients after treatment with nilotinib or imatinib (**Figures 5B, C**). The mRNA and protein levels of RAB32 were significantly increased following treatment with the miR-141-5p inhibitor (**Figures 5D, E**), and it was substantially decreased by the miR-141-5p mimics in K562 cells (**Figures 5F, G**). To confirm the effects of miR-141-5p on RAB32 mRNA level, we constructed RAB32 wt 3′UTR and transfected it with miR-141-5p mimics/NC into K562 cells. Cells cotransfected with the miR-141-5p mimics significantly suppressed RAB32 3′UTR activity compared to controls (**Figure 5H**). In conclusion, miR-141-5p suppressed the expression of RAB32 *via* specific binding to RAB32 mRNA 3′UTR.

### Downregulation of RAB32 Weakened K562 Cell Proliferation and Migration Abilities and Promoted Apoptosis

**Figure 6:**
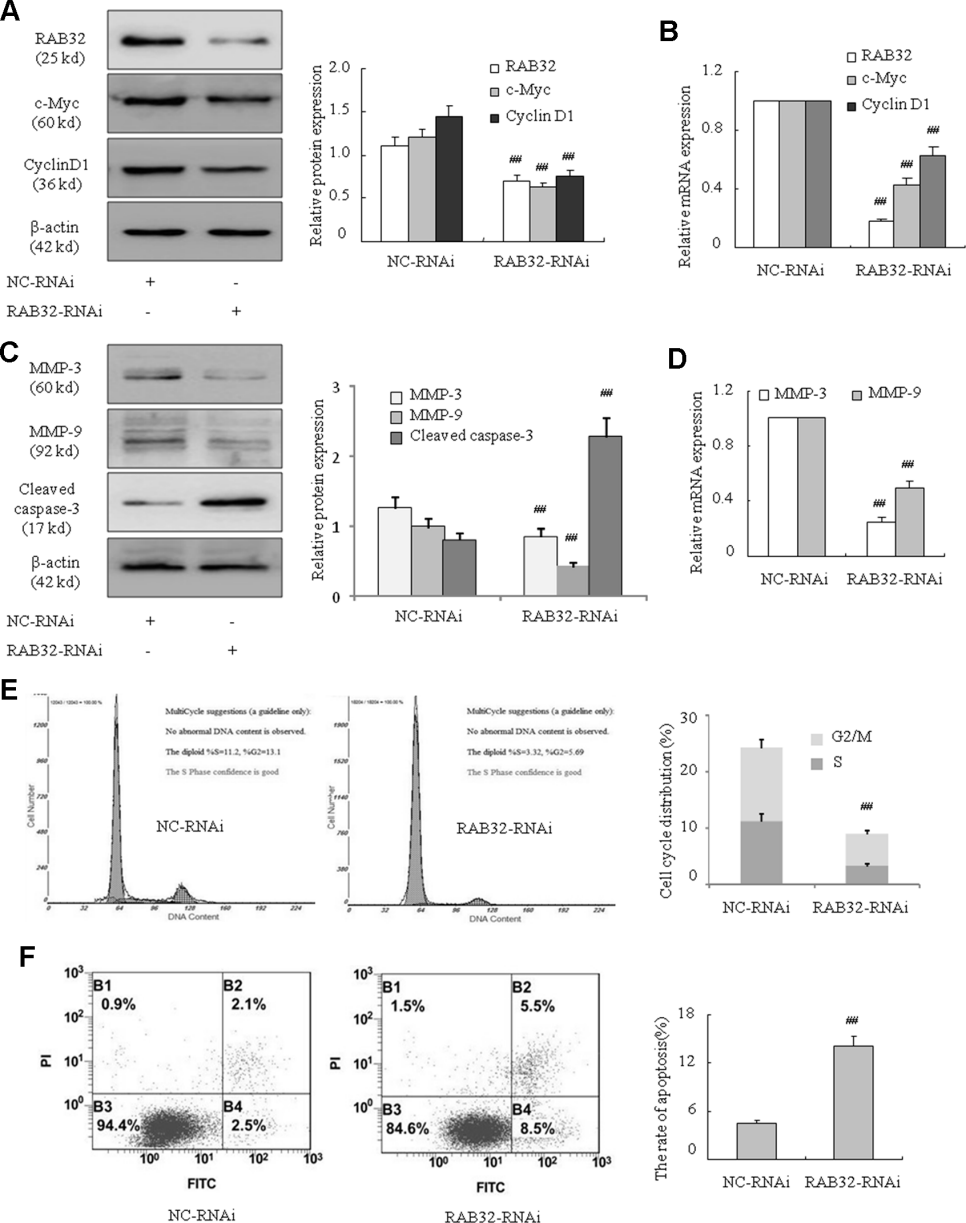
Downregulation of RAB32 reduced proliferation and migration and promoted apoptosis in K562 cells. **(A)** The protein expression of RAB32, c-Myc, and cyclin D1 were analyzed using Western blotting in K562 cells with RAB32-RNAi. **(B)** The mRNA expression of RAB32, c-Myc, and cyclin D1 were detected using q-PCR. **(C)** MMP-3, MMP-9, and cleaved caspase-3 protein levels were detected using Western blotting in K562 cells with RAB32-RNAi. **(D)** The relative mRNA expressions of MMP-3 and MMP-9 were detected using q-PCR. **(E)** Cell cycle of K562 cells was assessed using FACS analysis after incubation with RAB32-RNAi for 48 h. **(F)** After K562 cells were treated with RAB32-RNAi, cell apoptosis was analyzed using FACS analysis. All data are expressed as the means ± SD (^##^ *P* < 0.01 vs. NC group).

To further determine how RAB32 influenced the proliferation, migration, and apoptosis of CML cells, we used human RAB32-specific siRNA to knockdown RAB32 expression in K562 cells (**Figures 6A, B**). The results showed that c-Myc and cyclin D1 mRNA and protein levels were remarkably reduced in K562 cells after transfection with the RAB32 RNAi (**Figures 6A, B**). MMP-3 and MMP-9 (**Figures 6C, D**) were also decreased in the RAB32-RNAi in K562 cells. The level of cleaved caspase-3 was increased at the same time (**Figure 6C**). Similar to the miR-141-5p mimics, the RAB32-RNAi significantly reduced the proportions of S and G2/M phases in K562 cells (**Figure 6E**). Annexin V-FITC/PI staining showed that RAB32-RNAi promoted K562 cells apoptosis by down-regulating the RAB32 expression (**Figure 6F**). These results suggested that knockdown of RAB32 weakened K562 cell proliferation and migration abilities and promoted apoptosis.

### MiR-141-5p Affected K562 Cell Proliferation, Migration, and Apoptosis *via* RAB32

**Figure 7:**
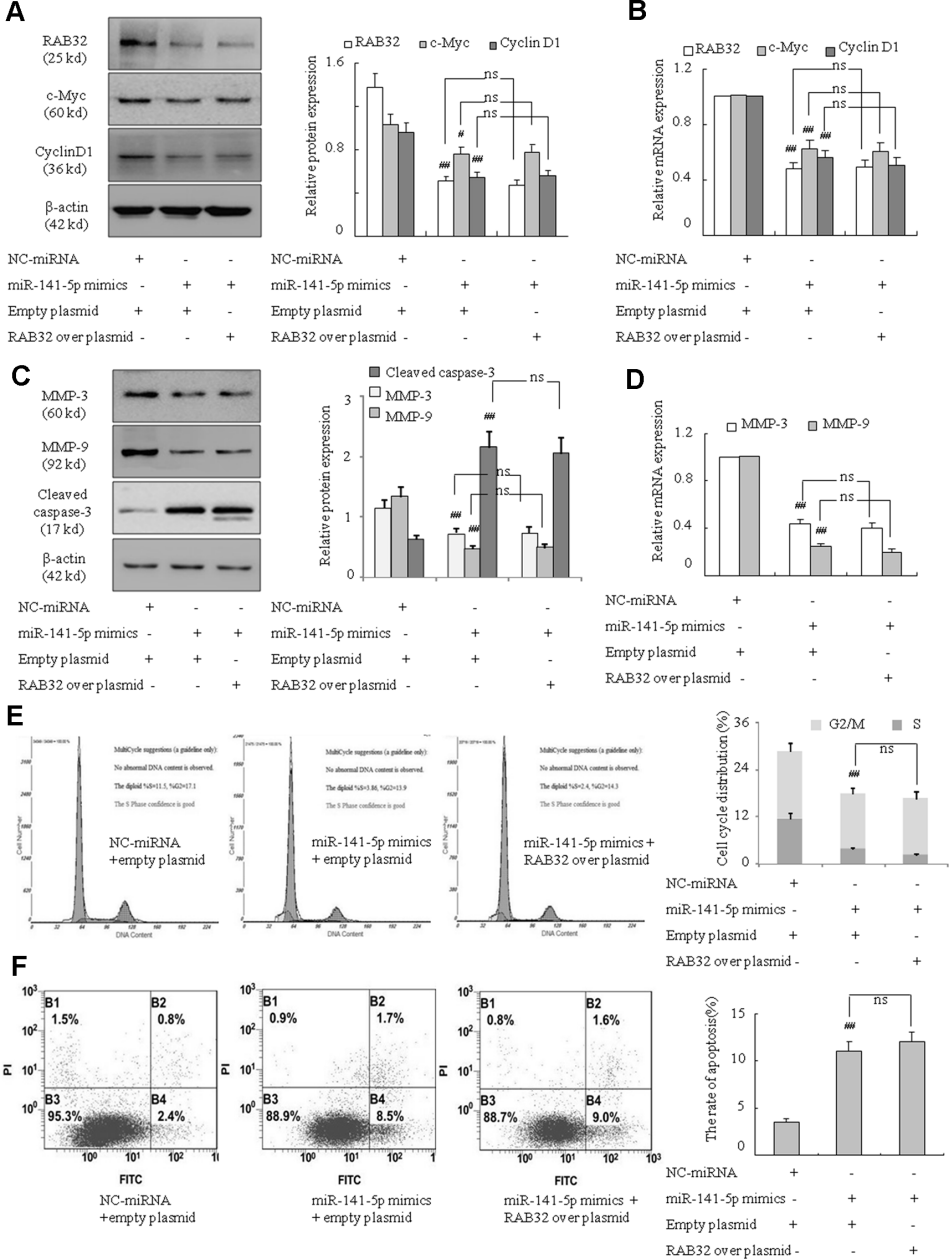
miR-141-5p modulated cell proliferation, migration and apoptosis *via* RAB32 in K562 cells. **(A)** The protein expression of RAB32, c-Myc, and cyclin D1 were analyzed using Western blotting in K562 cells. **(B)** The relative mRNA levels of RAB32, c-Myc, and cyclin D1 were detected using q-PCR. **(C)** The protein expression of MMP-3, MMP-9, and cleaved caspase-3 in K562 cells were determined using Western blotting. **(D)** The MMP-3 and MMP-9 mRNA levels were detected using q-PCR. **(E)** Cell cycle of K562 cells was assessed using FACS analysis after incubation with miR-141-5p mimics and RAB32-N1 for 48 h. **(F)** After K562 cells were treated with RAB32-N1 and miR-141-5p mimics, cell apoptosis was analyzed using FACS analysis. All data are expressed as the means ± SD (^#^ *P* < 0.05, ^##^ *P* < 0.01 vs. NC group; ns, *P* ≥ 0.005).

To further examine the underlying mechanism of miR-141-5p in association with RAB32 in CML, we assessed the role of the miR-141-5p mimics and overexpression of RAB32 on the proliferation, migration, and apoptosis in K562 cells. As shown in previous results, miR-141-5p mimics weakened the proliferation ability and promoted apoptosis of K562 cells. We also found that RAB32 was a direct target of miR-141-5p. Therefore, after treating with miR-141-5p mimics to upregulate the level of miR-141-5p, we investigated whether overexpression of RAB32 caused by RAB32 plasmid changed the proliferation, migration, and apoptosis conditions of K562 cells. **Figures 7A, B** showed that after treating with the miR-141-5p mimics and empty plasmid, c-Myc and cyclin D1 levels were significantly decreased as compared to the NC group. However, compared with the cotransfection group of miR-141-5p mimics and RAB32 overexpression plasmid (RAB32-N1), there was no significant difference in these two groups. MMP-3 and MMP-9 levels were also downregulated and cleaved caspase-3 level was upregulated by cotransfection with miR-141-5p mimics and empty plasmid. These levels were also unchanged as compared to the cotransfection group of miR-141-5p mimics and RAB32-N1 (**Figures 7C, D**). Cell cycle analysis indicated that S and G2/M phases of K562 cells were remarkably reduced after cotransfection with miR-141-5p mimics and empty plasmid, and it was unchanged compared with the cotransfection group of miR-141-5p mimics and RAB32-N1 (**Figure 7E**). The Annexin V-FITC/PI staining indicated that cotransfection cells with the miR-141-5p mimics and empty plasmid induced apoptosis of K562 cells, consistent with the cell cycle analysis results. The results of apoptosis were also unchanged compared with the cotransfection group of miR-141-5p mimics and RAB32-N1 in K562 cells (**Figure 7F**). The above results strongly suggested that RAB32-N1 did not alter the influence of miR-141-5p mimics on the proliferation, migration, and apoptosis of K562 cells. Therefore, we speculated that overexpression of miR-141-5p would inhibit RAB32 activity, despite the overexpression of RAB32 following transfection of the RAB32 over-producing plasmid. Therefore, miR-141-5p works to inhibit CML cell proliferation and migration and promotes cell apoptosis *via* targeting RAB32. Taken together, these results indicate that miR-141-5p plays an important role in the activation of K562 cells *in vitro* and may act as a tumor suppressor *via* targeting RAB32 in CML.

## Discussion

MiRNAs are involved in many biological processes, including cell proliferation, differentiation, and apoptosis, *via* the targeting of gene mRNA 3′UTR. Abnormal expression of certain miRNAs was observed in some cancers compared to normal tissues, such as endometrial carcinoma (Bao et al., 2013), breast cancer (Rhodes et al., 2015), ovarian cancer (Vilming Elgaaen et al., 2014), gastric cancer (Li et al., 2015), hepatocellular carcinoma (Yao et al., 2016), Hodgkin’s lymphoma (Paydas et al., 2016), thyroid cancer (Chen et al., 2017), acute promyelocytic leukemia (Garzon et al., 2007), bladder cancer (Wang et al., 2016), and chronic lymphoblastic leukemia (Calin et al., 2005). Various miRNAs may act as oncogenes or tumor suppressor genes according to their roles in tumorigenesis (Boufraqech, Klubo-Gwiezdzinska, & Kebebew, 2016). Many miRNAs play roles in CML, such as downregulation of miRNAs, including miR-124 (Jin et al., 2018), miR-199b (Joshi et al., 2014), miR-148b (Wang et al., 2013), miR-29b (Li et al., 2013), miR-320a (Xishan et al., 2015), and miR-203a (Chim et al., 2011), and the upregulation of miRNAs, such as miR-21 (Wang et al., 2015), miR-486-5p (Wang et al., 2015), and miR-130a (Suresh et al., 2011).

MiR-141 is widely expressed in many human malignant tumors and is highly expressed in tumors such as ovarian cancer (Mateescu et al., 2011), NSCLC tissues (Mei et al., 2014), nasopharyngeal cancer (Zhang et al., 2010), prostate cancer (Seol et al., 1996), thyroid papillary cancer (van Bokhoven et al., 2003), and colorectal cancer (Hu et al., 2010), but has low level in nasopharyngeal carcinoma (Li M. et al., 2019), hepatocellular carcinoma (Hou et al., 2019), esophageal cancer (Imanaka et al., 2011), non-small cell lung cancer (Li W. et al., 2019), breast cancer (Finlay-Schultz et al., 2015), and renal cell carcinoma (Yu et al., 2013). These results indicate that miR-141 plays a dual role of an oncogene or tumor suppressor by regulating target genes, which provides a new alternative for treatment in the course of different tumorigeneses and developments.

A previous study showed that the levels of miR-141-3p and 5p were observably reduced in CML samples. However, they were markedly upregulated in treated patients. Therefore, we supposed that miR-141 played a pivotal role in CML. We also proved that the levels of miR-141-3p and 5p were decreased in K562/G cells compared to K562 cells. MiR-141-5p showed a more remarkable change. Indeed, it is necessary to consider that miR-141-3p and 5p are closely related to the tumorigenesis of CML.

Considering that miR-141-5p was more significantly low-expressed in CML patients, miR-141-5p was selected for further studies of its role in the pathogenesis of K562 cells. C-Myc and cyclin D1 are important downstream signaling molecules in the cell proliferation pathway. C-Myc plays an important role in regulating the G-phase of the cell cycle as a proto-oncogene protein, and cyclin D1, acting on G1-phase, promotes G1/S phase transition and accelerates the process of cell cycle (Evan & Littlewood, 1993; Hoffman & Liebermann, 1998). We chose c-Myc and cyclin D1 as indicators of the proliferation of K562 cells. The results showed that suppression of miR-141-5p with an miR-141-5p inhibitor upregulated c-Myc and cyclin D1 in K562 cells. Migration is another important biological process in malignant tumors. Multiple studies confirmed that cell migration was related to matrix metalloproteinases (MMPs) (Wells et al., 2015; Tan et al., 2016). The MMP-3 and MMP-9 mRNA and protein levels were significantly upregulated after transfection with an miR-141-5p inhibitor. These results suggest that the down-regulation of miR-141-5p enhanced the proliferation and migration abilities of K562 cells. The uncontrolled proliferation of cells always corresponds with maladjusted cell cycle and shorter passage time (Scatena, 2012). Cell cycle analysis showed that miR-141-5p inhibitor led to the increased proportions of S phase and G2/M phase, which was consistent with the results of cell proliferation.

We also designed gain-of-function study by transfecting miR-141-5p mimics into K562 cells to simulate endogenous miRNAs and upregulate the level of miR-141-5p. The results showed that upregulation of miR-141-5p decreased c-Myc, cyclin D1, MMP-3, and MMP-9 expression and increased cleaved caspase-3 expression in K562 cells. Similarly, the percentages of K562 cells at S and G2/M stages were decreased, as shown in FACS. These results indicated that overexpression of miR-141-5p significantly inhibited proliferation and migration but enhanced apoptosis of K562 cells.

Cell proliferation and apoptosis maintain a dynamic balance in normal organisms. Excessive proliferation and apoptosis resistance may induce oncogenesis and the development of tumors (Scatena, 2012). Fei et al. (2012) found that MiR-181a induced apoptosis of the CML K562 cell line. Our study confirmed that the upregulation of miR-141-5p enhanced cell apoptosis in K562 cells with miR-141-5p mimics. In summary, overexpression of miR-141-5p remarkably inhibited proliferation and migration but enhanced apoptosis of K562 cells.

All of the above-mentioned experiments were performed *in vitro*. However, these results are not equivalent to the actual situation *in vivo*. Therefore, we constructed a xenograft model with nude mice to study tumor-related functions. Notably, our study demonstrated that tumor growth in the miR-141-5p mimics group was significantly slower compared to the control group, which is consistent with the results *in vitro*. The average tumor volume of the miR-141-5p mimics group was smaller after 21 days, and the average tumor weight of the miR-141-5p mimics group was also decreased compared to the control. Taken together, this evidence showed that overexpression of miR-141-5p suppressed tumor growth *in vivo*. These results indicated that miR-141-5p inhibited the proliferation and migration and enhanced apoptosis of K562 cells.

The present study used bioinformatics analysis and showed that RAB32 mRNA3′UTR was complementary with miR-141-5p. The potential target of miR-141-5p was identified as RAB32 *via* the construction and transfection of RAB32 3′UTR-WT and miR-141-5p mimics/NC into K562 cells, which caused fluorescence changes. Rab32-subfamily protein is a small molecule GTP-binding protein which has three members, including Rab29, Rab32, and Rab38. Rab29 shares 56% identities (77% similarities) to Rab32 within their G-domain fold (McGrath, Waschbusch, Baker, & Khan, 2019). Rab29 is a master regulator of LRRK2, controlling its activation, localization, and potentially biomarker phosphorylation (Purlyte et al., 2018). RAB38 regulates intracellular vesicular trafficking and some researchers have found that RAB38 may be an important prognostic factor in NSCLC, and serve a critical role in NSCLC-associated tumor metastasis (Hsieh et al., 2019). Recent studies demonstrated that the activation of RAB32 was related to ER stress protein in the MS brain, and high expression of RAB32 shortened the length of neuronal axons, changed the morphology of mitochondria, and accelerated apoptosis of nerve cells (Haile et al., 2017). Studies also suggested that RAB32 played an important role in the regulation of intracellular lipid metabolism (Schroeder et al., 2015; Li et al., 2016; Li et al., 2017). Our study treated the K562 cells with a miR-141-5p inhibitor, and the mRNA and protein expressions of RAB32 were upregulated in CML. Similarly, K562 cells transfected with miR-141-5p mimics significantly inhibited the RAB32 mRNA and protein levels compared to the control. We also found that RAB32 mRNA and protein levels were upregulated in CML samples. Conversely, RAB32 was markedly downregulated in patients under treatment with nilotinib or imatinib. The inhibition of RAB32 using RAB32-RNAi significantly suppressed the mRNA and protein expression of c-Myc, cyclin D1, MMP-3, and MMP-9 and promoted the protein expression of cleaved caspase-3 in K562 cells. Notably, RAB32-RNAi reduced the proportion of S-phase and G2/M-phase cells remarkably. Annexin V-FITC/PI staining revealed that RAB32-RNAi increased cell apoptosis, which means that knockdown of RAB32 induced apoptosis in K562 cells. These results indicated that proliferation and migration were inhibited, and apoptosis was enhanced in K562 cells after transfection with RAB32-RNAi. Notably, cotransfection with miR-141-5p mimics and empty plasmid led to a significant downregulation of the mRNA and protein levels of c-Myc, cyclin D1, MMP-3, and MMP-9 and upregulation of protein level of cleaved caspase-3 compared to the NC group. Cell cycle analysis also indicated that cotransfection with miR-141-5p mimics and empty plasmid obviously reduced S- and G2/M-phase cell proportions. Annexin V-FITC/PI staining also showed that cotransfection with miR-141-5p mimics and empty plasmid promoted apoptosis of K562 cells. However, there was no obvious difference when we cotransfected miR-141-5p and RAB32-N1. Therefore, miR-141-5p inhibited cell proliferation and migration and enhanced apoptosis of K562 cells *via* the targeting of RAB32.

## Conclusions

In conclusion, our study demonstrated that miR-141-5p played an important role in the activation of K562 cells and may act as a tumor suppressor *via* the targeting of RAB32 in CML. Therefore, miR-141-5p may provide new clues for the diagnosis and novel targeted therapy of CML.

## Data Availability Statement

All datasets generated for this study are included in the article.

## Ethics Statement

The Medical Ethics and Human Clinical Trial Committee of Anhui Medical University approved the experiment. The animals had ad libitum access to food and water at stable room temperature and humidity environment according to the Guide for the Care and Use of Laboratory Animals. All of the research subjects volunteered to donate their blood samples for the research.

## Author Contributions

JB, XL, and YL carried out the experiments, JB and XL analyzed the data, and JB interpreted the results and wrote the manuscript. CH and XM helped design the experiments and prepare the figures. JL designed the study and the experiments, and reviewed the manuscript. All authors have read and approved the final manuscript.

## Funding

This study was supported by the National Science Foundation of China (grant number 81770609).

## Conflict of Interest

The authors declare that the research was conducted in the absence of any commercial or financial relationships that could be construed as a potential conflict of interest.

## Abbreviations

CML, chronic myeloid leukemia; BCR/ABL, breakpoint cluster region/Abelson oncogene; Ph, Philadelphia chromosome; TKI, tyrosine kinase inhibitor; miRNA, MicroRNA; 3′-UTR, 3′-untranslated region; mRNA, messenger RNA; RNAi (siRNA), small interfering RNA; MMP, matrix metalloproteinases.

## References

[B1] Bao W. Wang H. H. Tian F. J. He X. Y. Qiu M. T. Wang J. Y. (2013). A TrkB-STAT3-miR-204-5p regulatory circuitry controls proliferation and invasion of endometrial carcinoma cells. Mol. Cancer 12, 155. 10.1186/1476-4598-12-155 24321270 PMC3879200

[B2] Boufraqech M. Klubo-Gwiezdzinska J. Kebebew E. (2016). MicroRNAs in the thyroid. Best Pract. Res. Clin. Endocrinol. Metab. 30 (5), 603–619. 10.1016/j.beem.2016.10.001 27923454 PMC5147609

[B3] Calin G. A. Ferracin M. Cimmino A. Di Leva G. Shimizu M. Wojcik S. E. (2005). A MicroRNA signature associated with prognosis and progression in chronic lymphocytic leukemia. N Engl. J. Med. 353 (17), 1793–1801. 10.1056/NEJMoa050995 16251535

[B4] Chen Y. Zhang S. Zhao R. Zhao Q. Zhang T. (2017). Upregulated miR-9-3p promotes cell growth and inhibits apoptosis in medullary thyroid carcinoma by targeting BLCAP. Oncol. Res. 25 (8), 1215–1222. 10.3727/096504016X14791715355957 27938505 PMC7841133

[B5] Chim C. S. Wong K. Y. Leung C. Y. Chung L. P. Hui P. K. Chan S. Y. (2011). Epigenetic inactivation of the hsa-miR-203 in haematological malignancies. J. Cell Mol. Med. 15 (12), 2760–2767. 10.1111/j.1582-4934.2011.01274.x 21323860 PMC4373446

[B6] Evan G. I. Littlewood T. D. (1993). The role of c-myc in cell growth. Curr. Opin. Genet. Dev. 3 (1), 44–49. S0959-437X(05)80339-9 8453273 10.1016/s0959-437x(05)80339-9

[B7] Fei J. Li Y. Zhu X. Luo X. (2012). miR-181a post-transcriptionally downregulates oncogenic RalA and contributes to growth inhibition and apoptosis in chronic myelogenous leukemia (CML). PloS One 7 (3), e32834. 10.1371/journal.pone.0032834 22442671 PMC3307705

[B8] Finlay-Schultz J. Cittelly D. M. Hendricks P. Patel P. Kabos P. Jacobsen B. M. (2015). Progesterone downregulation of miR-141 contributes to expansion of stem-like breast cancer cells through maintenance of progesterone receptor and Stat5a. Oncogene 34 (28), 3676–3687. 10.1038/onc.2014.298 25241899 PMC4369481

[B9] Garzon R. Pichiorri F. Palumbo T. Visentini M. Aqeilan R. Cimmino A. (2007). MicroRNA gene expression during retinoic acid-induced differentiation of human acute promyelocytic leukemia. Oncogene 26 (28), 4148–4157. 10.1038/sj.onc.1210186 17260024

[B10] Haile Y. Deng X. Ortiz-Sandoval C. Tahbaz N. Janowicz A. Lu J. Q. (2017). Rab32 connects ER stress to mitochondrial defects in multiple sclerosis. J. Neuroinflammation 14 (1), 19. 10.1186/s12974-016-0788-z 28115010 PMC5260063

[B11] Hilmarsdottir B. Briem E. Bergthorsson J. T. Magnusson M. K. Gudjonsson T. (2014). Functional role of the microRNA-200 family in breast morphogenesis and neoplasia. Genes (Basel) 5 (3), 804–820. 10.3390/genes5030804 25216122 PMC4198932

[B12] Hoffman B. Liebermann D. A. (1998). The proto-oncogene c-myc and apoptosis. Oncogene 17 (25), 3351–3357. 10.1038/sj.onc.1202592 9916997

[B13] Hou X. Yang L. Jiang X. Liu Z. Li X. Xie S. (2019). Role of microRNA-141-3p in the progression and metastasis of hepatocellular carcinoma cell. Int. J. Biol. Macromol. 128, 331–339. 10.1016/j.ijbiomac.2019.01.144 30695725

[B14] Hsieh J. J. Hou M. M. Chang J. W. Shen Y. C. Cheng H. Y. Hsu T. (2019). RAB38 is a potential prognostic factor for tumor recurrence in non-small cell lung cancer. Oncol. Lett. 18 (3), 2598–2604. 10.3892/ol.2019.10547 31452745 PMC6676642

[B15] Hu M. Xia M. Chen X. Lin Z. Xu Y. Ma Y. (2010). MicroRNA-141 regulates Smad interacting protein 1 (SIP1) and inhibits migration and invasion of colorectal cancer cells. Dig. Dis. Sci. 55 (8), 2365–2372. 10.1007/s10620-009-1008-9 19830559

[B16] Imanaka Y. Tsuchiya S. Sato F. Shimada Y. Shimizu K. Tsujimoto G. (2011). MicroRNA-141 confers resistance to cisplatin-induced apoptosis by targeting YAP1 in human esophageal squamous cell carcinoma. J. Hum. Genet. 56 (4), 270–276. 10.1038/jhg.2011.1 21289630

[B17] Jabbour E. Kantarjian H. (2014). Chronic myeloid leukemia: 2014 update on diagnosis, monitoring, and management. Am. J. Hematol. 89 (5), 547–556. 10.1002/ajh.23691 24729196

[B18] Jin J. Yao J. Yue F. Jin Z. Li D. Wang S. (2018). Decreased expression of microRNA-214 contributes to imatinib mesylate resistance of chronic myeloid leukemia patients by upregulating ABCB1 gene expression. Exp. Ther. Med. 16 (3), 1693–1700. 10.3892/etm.20186404 30186389 PMC6122133

[B19] Joshi D. Chandrakala S. Korgaonkar S. Ghosh K. Vundinti B. R. (2014). Down-regulation of miR-199b associated with imatinib drug resistance in 9q34.1 deleted BCR/ABL positive CML patients. Gene 542 (2), 109–112. 10.1016/j.gene.2014.03.049 24680705

[B20] Kim J. S. Kurie J. M. Ahn Y. H. (2015). BMP4 depletion by miR-200 inhibits tumorigenesis and metastasis of lung adenocarcinoma cells. Mol. Cancer 14, 173. 10.1186/s12943-015-0441-y 26395571 PMC4580148

[B21] Li Y. Wang H. Tao K. Xiao Q. Huang Z. Zhong L. (2013). MiR-29b suppresses CML cell proliferation and induces apoptosis via regulation of BCR/ABL1 protein. Exp. Cell Res. 319 (8), 1094–1101. 10.1016/j.yexcr.2013.02.002 23428668

[B22] Li H. L. Xie S. P. Yang Y. L. Cheng Y. X. Zhang Y. Wang J. (2015). Clinical significance of upregulation of mir-196a-5p in gastric cancer and enriched KEGG pathway analysis of target genes. Asian Pac. J. Cancer Prev. 16 (5), 1781–1787. 10.7314/apjcp.2015.16.5.1781 25773825

[B23] Li Z. Schulze R. J. Weller S. G. Krueger E. W. Schott M. B. Zhang X. (2016). A novel Rab10-EHBP1-EHD2 complex essential for the autophagic engulfment of lipid droplets. Sci. Adv. 2 (12), e1601470. 10.1126/sciadv.1601470 28028537 PMC5161429

[B24] Li C. Luo X. Zhao S. Siu G. K. Liang Y. Chan H. C. (2017). COPI-TRAPPII activates Rab18 and regulates its lipid droplet association. EMBO J. 36 (4), 441–457. 10.15252/embj.201694866 28003315 PMC5694949

[B25] Li M. Liu Y. Wei Y. Wu C. Meng H. Niu W. (2019). Zinc-finger protein YY1 suppresses tumor growth of human nasopharyngeal carcinoma by inactivating c-Myc-mediated microRNA-141 transcription. J. Biol. Chem. 294 (15), 6172–6187. 10.1074/jbc.RA118.006281RA118.006281 30718276 PMC6463721

[B26] Li W. Cui Y. Wang D. Wang Y. Wang L. (2019). MiR-141-3p functions as a tumor suppressor through directly targeting ZFR in non-small cell lung cancer. Biochem. Biophys. Res. Commun. 509 (3), 647–656. 10.1016/j.bbrc.2018.12.089 30611568

[B27] Mateescu B. Batista L. Cardon M. Gruosso T. de Feraudy Y. Mariani O. (2011). miR-141 and miR-200a act on ovarian tumorigenesis by controlling oxidative stress response. Nat. Med. 17 (12), 1627–1635. 10.1038/nm2512 22101765

[B28] McGrath E. Waschbusch D. Baker B. M. Khan A. R. (2019). LRRK2 binds to the Rab32 subfamily in a GTP-dependent manner via its armadillo domain. Small GTPases, 25, 1–14. 10.1080/21541248.2019.1666623 PMC7849779 31552791

[B29] Mei Z. He Y. Feng J. Shi J. Du Y. Qian L. (2014). MicroRNA-141 promotes the proliferation of non-small cell lung cancer cells by regulating expression of PHLPP1 and PHLPP2. FEBS Lett. 588 (17), 3055–3061. 10.1016/j.febslet.2014.06.020 24945731

[B30] Men D. Liang Y. Chen L. (2014). Decreased expression of microRNA-200b is an independent unfavorable prognostic factor for glioma patients. Cancer Epidemiol. 38 (2), 152–156. 10.1016/j.canep.2014.01.003 24559637

[B31] Patnaik M. M. Parikh S. A. Hanson C. A. Tefferi A. (2014). Chronic myelomonocytic leukaemia: a concise clinical and pathophysiological review. Br. J. Haematol. 165 (3), 273–286. 10.1111/bjh.12756 24467717

[B32] Paydas S. Acikalin A. Ergin M. Celik H. Yavuz B. Tanriverdi K. (2016). Micro-RNA (miRNA) profile in Hodgkin lymphoma: association between clinical and pathological variables. Med. Oncol. 33 (4), 34. 10.1007/s12032-016-0749-5 26951445

[B33] Pereira-Leal J. B. Seabra M. C. (2000). The mammalian Rab family of small GTPases: definition of family and subfamily sequence motifs suggests a mechanism for functional specificity in the Ras superfamily. J. Mol. Biol. 301 (4), 1077–1087. 10.1006/jmbi.20004010 10966806

[B34] Prashar A. Schnettger L. Bernard E. M. Gutierrez M. G. (2017). Rab GTPases in immunity and inflammation. Front. Cell Infect. Microbiol. 7, 435. 10.3389/fcimb.2017.00435 29034219 PMC5627064

[B35] Purlyte E. Dhekne H. S. Sarhan A. R. Gomez R. Lis P. Wightman M. (2018). Rab29 activation of the Parkinson’s disease-associated LRRK2 kinase. EMBO J. 37 (1), 1–18. 10.15252/embj.201798099 29212815 PMC5753036

[B36] Rhodes L. V. Martin E. C. Segar H. C. Miller D. F. Buechlein A. Rusch D. B. (2015). Dual regulation by microRNA-200b-3p and microRNA-200b-5p in the inhibition of epithelial-to-mesenchymal transition in triple-negative breast cancer. Oncotarget 6 (18), 16638–16652. 10.18632/oncotarget3184 26062653 PMC4599295

[B37] San Jose-Eneriz E. Roman-Gomez J. Jimenez-Velasco A. Garate L. Martin V. Cordeu L. (2009). MicroRNA expression profiling in imatinib-resistant chronic myeloid leukemia patients without clinically significant ABL1-mutations. Mol. Cancer 8, 69. 10.1186/1476-4598-8-69 19723306 PMC2743636

[B38] Scatena R. (2012). Mitochondria and cancer: a growing role in apoptosis, cancer cell metabolism and dedifferentiation. Adv. Exp. Med. Biol. 942, 287–308. 10.1007/978-94-007-2869-1_13 22399428

[B39] Schroeder B. Schulze R. J. Weller S. G. Sletten A. C. Casey C. A. McNiven M. A. (2015). The small GTPase Rab7 as a central regulator of hepatocellular lipophagy. Hepatology 61 (6), 1896–1907. 10.1002/hep.27667 25565581 PMC4441591

[B40] Seol W. Choi H. S. Moore D. D. (1996). An orphan nuclear hormone receptor that lacks a DNA binding domain and heterodimerizes with other receptors. Science 272 (5266), 1336–1339. 10.1126/science.272.5266.1336 8650544

[B41] Spizzo R. Nicoloso M. S. Croce C. M. Calin G. A. (2009). SnapShot: microRNAs in cancer. Cell 137 (3), 586–586 e581. 10.1016/j.cell.2009.04.040 19410551

[B42] Suresh S. McCallum L. Lu W. Lazar N. Perbal B. Irvine A. E. (2011). MicroRNAs 130a/b are regulated by BCR-ABL and downregulate expression of CCN3 in CML. J. Cell Commun. Signal 5 (3), 183–191. 10.1007/s12079-011-0139-x 21638198 PMC3145871

[B43] Tan C. Qiao F. Wei P. Chi Y. Wang W. Ni S. (2016). DIXDC1 activates the Wnt signaling pathway and promotes gastric cancer cell invasion and metastasis. Mol. Carcinog 55 (4), 397–408. 10.1002/mc.22290 25648220

[B44] van Bokhoven A. Varella-Garcia M. Korch C. Johannes W. U. Smith E. E. Miller H. L. (2003). Molecular characterization of human prostate carcinoma cell lines. Prostate 57 (3), 205–225. 10.1002/pros.10290 14518029

[B45] Vilming Elgaaen B. Olstad O. K. Haug K. B. Brusletto B. Sandvik L. Staff A. C. (2014). Global miRNA expression analysis of serous and clear cell ovarian carcinomas identifies differentially expressed miRNAs including miR-200c-3p as a prognostic marker. BMC Cancer 14, 80. 10.1186/1471-2407-14-80 24512620 PMC3928323

[B46] Wang L. Liu Y. Beier U. H. Han R. Bhatti T. R. Akimova T. (2013). Foxp3+ T-regulatory cells require DNA methyltransferase 1 expression to prevent development of lethal autoimmunity. Blood 121 (18), 3631–3639. 10.1182/blood-2012-08-451765 23444399 PMC3643763

[B47] Wang L. S. Li L. Chu S. Shiang K. D. Li M. Sun H. Y. (2015). MicroRNA-486 regulates normal erythropoiesis and enhances growth and modulates drug response in CML progenitors. Blood 125 (8), 1302–1313. 10.1182/blood-2014-06-581926 25515961 PMC4335083

[B48] Wang W. Z. Pu Q. H. Lin X. H. Liu M. Y. Wu L. R. Wu Q. Q. (2015). Silencing of miR-21 sensitizes CML CD34+ stem/progenitor cells to imatinib-induced apoptosis by blocking PI3K/AKT pathway. Leuk. Res. 39 (10), 1117–1124. 10.1016/j.leukres.2015.07.008 26248946

[B49] Wang X. Liang Z. Xu X. Li J. Zhu Y. Meng S. (2016). miR-148a-3p represses proliferation and EMT by establishing regulatory circuits between ERBB3/AKT2/c-myc and DNMT1 in bladder cancer. Cell Death Dis. 7 (12), e2503. 10.1038/cddis.2016.373 27906180 PMC5261009

[B50] Wells J. M. Gaggar A. Blalock J. E. (2015). MMP generated matrikines. Matrix Biol. 44-46, 122–129. 10.1016/j.matbio.2015.01.016 25636538 PMC4838901

[B51] Xishan Z. Ziying L. Jing D. Gang L. (2015). MicroRNA-320a acts as a tumor suppressor by targeting BCR/ABL oncogene in chronic myeloid leukemia. Sci. Rep. 5, 12460. 10.1038/srep12460 26228085 PMC4521206

[B52] Yao S. Tian C. Ding Y. Ye Q. Gao Y. Yang N. (2016). Down-regulation of Kruppel-like factor-4 by microRNA-135a-5p promotes proliferation and metastasis in hepatocellular carcinoma by transforming growth factor-beta1. Oncotarget 7 (27), 42566–42578. 10.18632/oncotarget9934 27302923 PMC5173156

[B53] Yu X. Y. Zhang Z. Liu J. Zhan B. Kong C. Z. (2013). MicroRNA-141 is downregulated in human renal cell carcinoma and regulates cell survival by targeting CDC25B. Onco. Targets Ther. 6, 349–354. 10.2147/OTT.S41343 23596351 PMC3627343

[B54] Zhang L. Deng T. Li X. Liu H. Zhou H. Ma J. (2010). microRNA-141 is involved in a nasopharyngeal carcinoma-related genes network. Carcinogenesis 31 (4), 559–566. 10.1093/carcin/bgp335 20053927

[B55] Zhou X. Wang Y. Shan B. Han J. Zhu H. Lv Y. (2015). The downregulation of miR-200c/141 promotes ZEB1/2 expression and gastric cancer progression. Med. Oncol. 32 (1), 428. 10.1007/s12032-014-0428-3 25502084

